# Disseminated Gastrointestinal Basidiobolomycosis: A Case Report with Review of Diagnostic Clues

**DOI:** 10.1155/2024/5741625

**Published:** 2024-08-28

**Authors:** Neda Soleimani, Mohammad Hossein Anbardar, Hamed Nikoupour, Faranak Derakhshan, Mojtaba Shafiekhani, Sahand Mohammadzadeh, Seyed Mohamad Sakhaei, Mahsa Farhadi

**Affiliations:** ^1^ Department of Pathology Shiraz Medical School Shiraz University of Medical Sciences, Shiraz, Iran; ^2^ Department of Pathology Shiraz Transplant Center Abu Ali Sina Hospital Shiraz University of Medical Sciences, Shiraz, Iran; ^3^ Shiraz Organ Transplant Center Abu-Ali Sina Hospital Shiraz University of Medical Sciences, Shiraz, Iran; ^4^ Department of Surgery Shiraz Transplant Center Abu Ali Sina Hospital Shiraz University of Medical Sciences, Shiraz, Iran; ^5^ Department of Clinical Pharmacy Faculty of Pharmacy, Shiraz University of Medical Sciences, Shiraz, Iran; ^6^ Department of Radiology Islamic Azad University, Sari Branch, Tehran, Iran

## Abstract

**Introduction:**

Basidiobolomycosis is a rare fungal infection caused by an environmental saprophyte, *Basidiobolus ranarum*. It usually presents as a chronic subcutaneous infection; however, few cases of gastrointestinal involvement have been reported. The exact transmission route of gastrointestinal cases is not clear, and diagnosis always requires a high index of suspicion because it tends to mimic other inflammatory and neoplastic conditions. *Case Report*. A 31-year-old immunocompetent woman presented with abdominal pain and an advanced colon mass. She was completely well until about 1.5 years ago, when she underwent bariatric surgery. One year after surgery, chronic abdominal pain developed. A colonoscopy showed an ulcerative lesion in the descending colon, and the biopsy was in favor of ulcerative colitis. Despite immunosuppressive treatment, there was no improvement, and with worsening symptoms, more investigations revealed advanced colon mass with entrapment of the stomach and pancreas. Colonic mucosa biopsy and trucut biopsy of the mass showed just necrosis and acute inflammation; thus, she underwent exploratory laparotomy with colectomy, partial gastrectomy, distal pancreatectomy, and left nephrectomy. On pathologic examination, there was granulomatous inflammation plus the Splendore–Hoeppli phenomenon around fungal hyphae, which was diagnostic for gastrointestinal basidiobolomycosis. Previous pathology slides were reviewed and revealed a tiny focus of basidiobolomycosis. After 6 months of treatment with itraconazole, she is relatively well without any clinical or radiologic abnormalities.

**Conclusion:**

Our case highlights the significance of suspicion for basidiobolomycosis in ulcerative and necrotic lesions with increased eosinophils, especially in the presence of abdominal mass and systemic eosinophilia.

## 1. Introduction

Basidiobolomycosis is a rare fungal infection caused by the fungus *Basidiobolus ranarum* of the Entomophthorales family. This fungus is an environmental saprophyte found in soil and decaying vegetable materials, especially in tropical and subtropical areas. Basidiobolomycosis mainly affects immunocompetent individuals as a chronic subcutaneous infection. Gastrointestinal involvement is extremely rare [1, 2]. The diagnosis of gastrointestinal basidiobolomycosis (GIB) is always confusing and requires a high index of suspicion because it tends to mimic other non-neoplastic and also neoplastic conditions, even on histopathologic evaluation. The exact transmission route is also not clear in some cases [2, 3]. In the reported case of GIB, misdiagnosis with inflammatory bowel disease led to disseminated visceral involvement.

## 2. Case Report

A 31-year-old woman from the north of Iran was transferred to Abu Ali Sina Hospital, Shiraz, Iran, to be evaluated for advanced colon mass. She was completely well until about 1.5 years ago, when she underwent bariatric surgery for morbid obesity (BMI: 50.1). One year after surgery, she presented with intermittent, generalized, and progressive abdominal pain and fever, which lasted for a month. There was no history of nausea, vomiting, or diarrhea.

**Figure 1:**
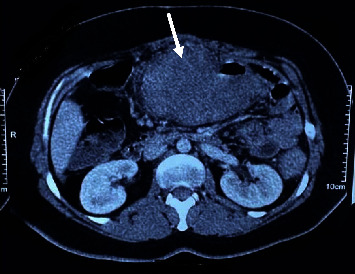
Abdominal CT scan with contrast shows a huge mass in the transverse colon.

A colonoscopy showed an ulcerative lesion in the descending colon, and a biopsy was in favor of ulcerative colitis. Despite treatment with immunosuppressant drugs, there was no improvement after 6 months, and with worsening of symptoms, more investigations by computed tomography (CT) revealed a large complex new malignant-looking abdominal mass encasing the transverse colon, stomach, tail of pancreas, and left kidney (Figure 1). A repeated colonoscopic biopsy and trucut biopsy of the mass showed ulceration, necrosis, and an acute inflammatory reaction.

**Figure 2:**
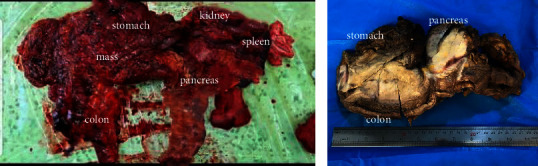
The gross morphology of the case shows extensive visceral involvement (a) in the operation room and (b) after formalin fixation at the pathology department.

A physical examination on admission showed a low-grade fever (37.9°C) and abdominal tenderness. Laboratory data showed mild leukocytosis, mild anemia, and eosinophilia with increased erythrocyte sedimentation rate (ESR) and C-reactive protein (CRP). Other laboratory tests, including urea, creatinine, glucose, and liver function tests, were within normal ranges. According to the clinical and radiologic data and also based on the previous histologic diagnosis of ulcerative colitis, she underwent exploratory laparotomy in our center with a preoperation diagnosis of advanced colon malignancy, which showed extensive involvement of the visceral organs (Figure 2(a)).

**Figure 3:**
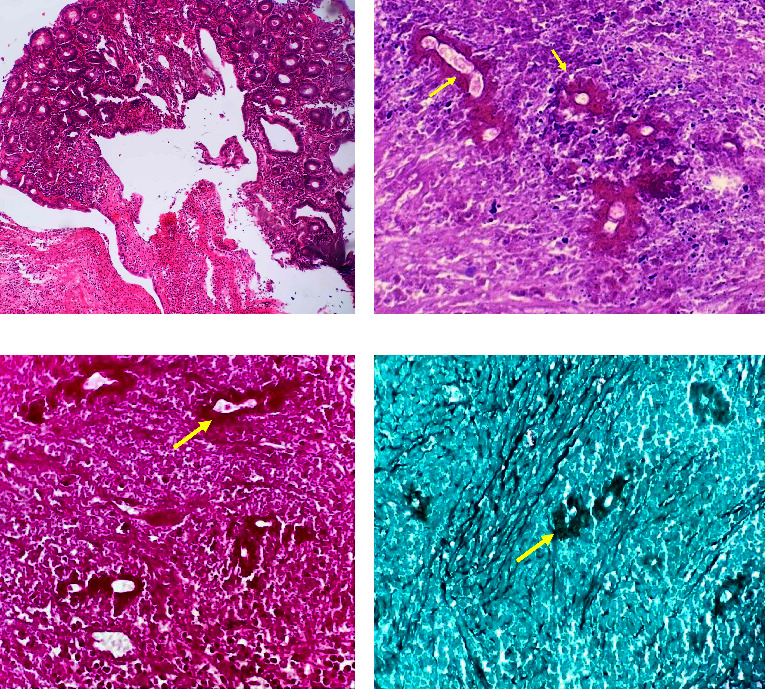
The microscopic view of the specimen. (a) Ulceration with normal overlying colon mucosa (hematoxylin and eosin ×100). (b) Necrosis with the Splendore–Hoeppli phenomenon (arrow) (hematoxylin and eosin ×400). (c) Splendore–Hoeppli phenomenon (arrow) (Periodic Acid-Schiff (PAS) ×400). (d) Splendore–Hoeppli phenomenon (arrow) (Grocott's Methenamine Silver (GMS) stain ×400).

The specimens of total colectomy, hemigastrectomy, distal pancreatectomy, splenectomy, and left nephrectomy were sent for pathologic examination. The macroscopic evaluation revealed two well-demarcated, rubbery, creamy masses. The largest mass was 17 × 15 × 10 cm found in the submucosa of the colon and pancreas with attachment to the stomach (Figure 2(b)). Histologic examination of the mentioned masses and pancreas revealed acute and chronic inflammatory changes with a predominantly granulomatous reaction and a rise in the eosinophilic count. Basidiobolomycosis infection was suspected based on macroscopic mass formation and the presence of broad, thin-walled, and aseptate hyphae, in addition to the Splendore–Hoeppli phenomenon (radiating intensely eosinophilic material) surrounding the fungal hyphae. Special stains for fungal elements, such as Periodic Acid-Schiff (PAS) and Grocott's Methenamine Silver (GMS), were also used to confirm the diagnosis (Figure 3). The colonic mucosa showed focal ulceration and activity without any evidence of chronic colitis. The specimen was sent to pathology in a 10% formalin solution to rule out cancer, but unfortunately, tissue culture was not performed because there was not enough suspicion for a fungal infection.

**Figure 4:**
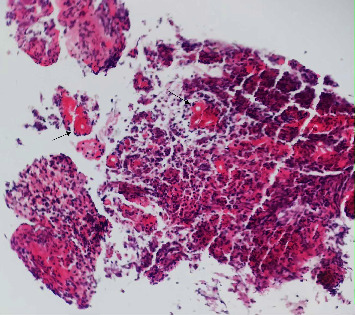
Review of the previous biopsy showed a tiny focus of the Splendore–Hoeppli phenomenon (arrows) (hematoxylin and eosin ×40).

The pathologic slides of the previous biopsies from another center were also reviewed and showed a tiny focus of the Splendore–Hoeppli phenomenon, which was missed due to the unfamiliarity of the pathologists with this clinical entity (Figure 4).

Intravenous antifungal therapy with fluconazole was started, but since the patient could not tolerate it and developed nausea, vomiting, and diarrhea after two days, it was replaced by oral itraconazole for 6 months. In a follow-up visit two weeks later, the patient showed improvement in her symptoms, and during the 6-month follow-up visits, no clinical or radiologic deficits were noted.  Table 1 shows the transition of laboratory data from the first day of admission to recovery time.

We requested and obtained informed consent from the patient for publishing the case report and the accompanying images. The research was approved by the Ethics Committee of Shiraz University of Medical Sciences (No. IR.SUMS.MED.REC.1400.433).

## 3. Discussion

We investigated the English literature in the period 1964–2021 via PubMed, Google, and Google Scholar twice, using the following search keywords: basidiobolomycosis and gastrointestinal and Iran.

*Basidiobolus ranarum* is a worldwide environmental saprophyte causing basidiobolomycosis fungal infection. The disease typically affects the skin and subcutaneous tissue, with rare gastrointestinal involvement [4–6].

A young Nigerian boy was the first known case of GIB, as described by Edington in 1964. Following that, the United States, Saudi Arabia, India, and Iran all had a small number of case reports. All previous Iranian case reports were from the south of the country [7, 8]; our case, however, was from a northern province, and to the best of our knowledge, no case of GIB had been reported from that region in English literature until now.

Similar to our situation, every case that has been published has been immunocompetent and has no clear risk factors. Although the patient in our case was an adult female, most cases that have been documented were young boys [8, 9].

Ingestion of infected foods is considered the main route of infection, and disturbing gastric mucosal defense due to the use of ranitidine and smoking may facilitate fungal infection after gastric entrance. Using contaminated papers for cleaning the skin (e.g., toilet papers) and exposure in the work environment are other proposed theories [10–12]. So far, few cases like ours have been reported with a history of previous surgery, including herniorrhaphy, appendectomy, and laparotomy for peptic ulcer perforation [11, 13, 14].

GIB mostly involves the colon. Abdominal pain, fever, diarrhea or constipation, weight loss, and abdominal mass are the primary clinical manifestations. Cases have also been reported with retroperitoneal fibrosis and ileocecal intussusception [8, 15]. In most cases, these nonspecific symptoms and signs result in a misdiagnosis of malignancy, inflammatory bowel disease (such as Crohn's disease or ulcerative colitis), tuberculosis, sarcoidosis, etc. In a review by Al Jarie, none of the reported cases had a diagnostic endoscopic biopsy. This misdiagnosis typically causes a delay in diagnosis, followed by an increase in morbidity and mortality [4–6, 16, 17].

Peripheral blood eosinophilia is a common laboratory finding, as increased ESR, anemia, and thrombocytosis [18–20]. However, the diagnosis of GIB depends on the detection of *B. ranarum* on a morphological, microbiological (tissue culture), or molecular basis (PCR). The best results are obtained by using DNA extracted from formalin-fixed paraffin-embedded lesional tissues. However, this method is not widely used by many health institutions, as well as our center, due to the rarity of basidiobolomycosis infections. Culture on Sabouraud agar at 20–30°C with incubation for 2-3 days is the gold standard for definitive diagnosis [20–22]. However, in most reported cases, similar to ours, the patients had undergone surgical resection with the clinical impression of mass, and therefore the specimens have been transferred to the laboratories in formalin and no cultures have been performed [21, 23].

The main histologic findings include mixed inflammatory cells, especially eosinophilic infiltration, granulomatous reactions, broad, thin-walled, and aseptate hyphae, and Splendore–Hoeppli phenomenon (radiating intensely eosinophilic material) around fungal hyphae. Because the Splendore–Hoeppli phenomenon can be caused by a variety of microorganisms and inert substances, special staining for fungal elements, such as PAS and GMS stains, or culture should be used to confirm the presence of fungus [8, 10, 20]. The other fungal infections with similar morphology to *B. ranarum* are *Conidiobolus coronatus, Conidiobolus incongruous*, and *Pythium*. They are separated from *B. ranarum* by the fact that these fungi affect the head and neck region in immunocompromised patients. Visceral involvement by these fungi has not been reported [21, 22].

Among variable differential diagnoses, inflammatory bowel disease is the most critical one. Severe colitis in inflammatory bowel disease is usually accompanied by architectural distortion, ulceration, and mixed inflammatory cell infiltration. Thus, in the absence of a high clinical suspicion for basidiobolomycosis and looking for the fungus, small biopsy specimens without granulomatous reaction may easily be missed as ulcerative colitis. Moreover, superficial intestinal mucosal biopsies in GIB can show nonspecific chronic inflammation [24]. Unfortunately, there is no definite confirmatory test for inflammatory bowel disease, and treatment with immunosuppressive therapy may lead to disseminated fungal infection, as in our case.

Surgical resection of the infected bowel with a wide margin, followed by 6–12 months of systemic antifungal therapy, is the treatment of choice [8, 24, 25]. Itraconazole is the most commonly used antifungal medication, followed by amphotericin, ketoconazole, and voriconazole. *B. ranarum* is intrinsically resistant to amphotericin B. voriconazole is a useful and generally well-tolerated drug for treating uncommon fungal infections and should be considered a backup plan in patients with no effective outcomes after being treated with other antifungal drugs. However, with the increasing likelihood of resistant strains, culture and sensitivity tests are necessary to identify the most effective antifungal treatment plan for each individual situation [26–28]. Long-term medical treatment and follow-up are advised to prevent the recurrence, and the disseminated cases should be treated with debridement and antifungal therapy [25, 29, 30].

Our patient was initially diagnosed with ulcerative colitis based on clinical presentation and colonoscopic biopsies and was later thought to have advanced colon cancer according to the radiologic assessment. If the pathologists were educated about the histologic characteristics of GIB or observed increased ESR, eosinophilia, and significant eosinophil infiltration in the colonic tissue, the initial colon biopsy could be suggestive of the disease and prevent misdiagnosis and additional visceral involvement. Moreover, it is recommended that the Middle Eastern countries, who comprise the majority of GIB patients, furnish their laboratories for relevant PCR and fungal culture.

## 4. Conclusion

Although a high index of suspicion is required for preoperative diagnosis of GIB, some clinical and paraclinical tips, such as peripheral eosinophilia and abdominal mass formation with eosinophilic infiltration in the context of previous intraabdominal intervention, should raise suspicion for the disease and, if put together correctly, could help to solve the puzzle and enable diagnosis. As in our situation, a delayed diagnosis might lead to the infection spreading and necessitating advanced surgery.

## Figures and Tables

**Table 1:** The initial and posttreatment results (after 8 weeks) of the laboratory tests.

Parameter	Initial results	Posttreatment results	Reference range (units)
WBC (eosinophil%)	15.4 (18%)	7.8 (5%)	4.5–11 (×10^3^/*μ*L) (3–5%)
Hemoglobin	11	13.2	12–16 (g/dL)
Platelet	423	275	150–450 (×10^3^/*μ*L)
ESR	44	12	0–20 (mm/Hr)
CRP	48	<6	<6 (mg/L)
AST	34	28	3–40 (IU/L)
ALT	27	22	3–40 (IU/L)
ALP	282	160	80–306 (IU/L)
LDH	390	268	200–400 (IU/L)
FBS	98	86	70–100 (mg/dL)
Urea	28	23	15–40 (mg/dL)
Creatinine	0.7	0.8	0.5–1.3 (mg/dL)

WBC: white blood cell; ESR: erythrocyte sedimentation rate; CRP: C-reactive protein; AST: aspartate aminotransferase; ALT: alanine aminotransferase; ALP: alkaline phosphatase; LDH: lactate dehydrogenase; FBS: fasting blood sugar.

## Data Availability

All data generated or analysed during this study are included in this published article.

## Abbreviations

CRP: C-reactive protein
CT: Computed tomography
ESR: Erythrocyte sedimentation rate
GIB: Gastrointestinal basidiobolomycosis.
